# Single cell segmental aneuploidy detection is compromised by S phase

**DOI:** 10.1186/1755-8166-7-46

**Published:** 2014-07-11

**Authors:** Eftychia Dimitriadou, Niels Van der Aa, Jiqiu Cheng, Thierry Voet, Joris R Vermeesch

**Affiliations:** 1 Laboratory for Cytogenetics and Genome Research, Department of Human Genetics, KU Leuven, Leuven 3000, Belgium; 2 Laboratory of Reproductive Genomics, Department of Human Genetics, KU Leuven, Leuven 3000, Belgium

## Abstract

**Background:**

Carriers of balanced translocations are at high risk for unbalanced gametes which can result in recurrent miscarriages or birth defects. Preimplantation genetic diagnosis (PGD) is often offered to select balanced embryos. This selection is currently mainly performed by array CGH on blastomeres. Current methodology does not take into account the phase of the cell cycle, despite the variable copy number status of different genomic regions in S phase.

**Results:**

Cell lines derived from 3 patients with different chromosomal imbalances were used to evaluate the accuracy of single cell array CGH. The different cell cycle phases were sorted by flow cytometry and 10 single cells were picked per cell line per cell cycle phase, whole genome amplified and analyzed by BAC arrays, the most commonly used platform for PGD purposes. In contrast to G phase, where the imbalances were efficiently identified, less than half of the probes in the regions of interest indicated the presence of the aberration in 17 S-phase cells, resulting in reduced accuracy.

**Conclusions:**

The results demonstrate that the accuracy to detect segmental chromosomal imbalances is reduced in S-phase cells, which could be a source of misdiagnosis in PGD. Hence, the cell cycle phase of the analyzed cell is of great importance and should be taken into account during the analysis. This knowledge may guide future technological improvements.

## Background

Up to 15% of the couples confront fertility problems and 1% of the couples attempting to conceive a child experience recurrent miscarriage (RM), defined as the loss of at least three consecutive pregnancies [1]. In approximately 3-6% of couples with RM the cause is a balanced chromosomal rearrangement carried by one of the partners [2,3], which may result in segmental aneuploid conceptions incompatible with life [4]. In order to avoid miscarriages or the possibility of an affected child, preimplantation genetic diagnosis (PGD) can be offered to select those embryos which are chromosomally balanced. PGD requires the use of assisted reproduction technology (ART) and it has already been used since the beginning of the ‘90s, initially applied for monogenic diseases [5] and shortly after for chromosomal rearrangements [6]. At the same time, preimplantation genetic screening (PGS) to identify numerical chromosome aberrations was applied to embryos from women of advanced age (advanced maternal age, AMA) and those who had suffered repeated implantation failure (RIF) and RM [7,8].

Overall, during the 10 years of data collection by the European Society of Human Reproduction and Embryology (ESHRE), there have been 27,630 cycles to oocyte retrieval (OR) reported, that resulted in 202,357 fertilized oocytes and the transfer of 35,944 embryo. For 16% of these cycles the indication was chromosomal abnormalities [9].

Initially, fluorescence *in situ* hybridization (FISH) was used for chromosome analysis, but technical difficulties and restrictions associated to this method lately lead to the introduction of array based comparative genomic hybridization (aCGH), which allows the simultaneous analysis of all 24 chromosomes at once, and thought to be a more robust and reliable test. The ESHRE PGS task force has completed pilots regarding the use of aCGH for PGD purposes [10,11], and a multi-center randomized controlled trial has been set up. Bacterial artificial chromosome (BAC) aCGH-based PGD is currently by far the most commonly used method and regarded as the gold standard. As it offers a resolution down to 2.5 Mb in single cells, the majority of translocations can be analyzed and even for translocations with smaller exchanged fragments, provided that three out of the four fragments of interest are above the resolution limit, the unbalanced products can be detected [12-14].

SNP arrays [15-19] and even single-cell sequencing [20-23] seem to be promising, but they are still being developed and need to be further optimized and validated, while issues regarding the ethical concerns related to the large amount of genetic information that will be available from each embryo still need to be addressed.

Although the literature abounds in studies evaluating different PGD approaches, none of the current methods take into consideration the cell cycle phase of the cells used for the analysis. It is known that the DNA of the S-phase cell is progressively replicated from multiple origins of replication (ORIs) [24] and log2 ratios of single S-phase cells follow the patterns of early and late replication domains [25]. Different replicons are organized in replication domains that follow similar replication timing, forming early and late replication domains [26]. But the firing of the different ORIs occurs stochastically. Hence, at a given time point the genetic copy number (CN) profiling of an S-phase cell will demonstrate different loci with CN status of 2, 3 or 4, depending on the replication status of this specific locus. It has also been proposed that the DNA replication process could result in errors of interpretation (misdiagnosis) in proliferating cells by FISH [27].

It still remains unanswered, though, to what extent this oscillation can influence the accuracy of CN profiling in single cells and to what extent the sensitivity and the specificity of CN determination would be affected. On one hand, the increased number of false positive calls could lead to misdiagnosis if they are falsely interpreted as real genuine structural aberrations. On top of that, some aberrations could be masked due to the high S-phase genome fluctuation and missed when S-phase cells are analyzed by aCGH, thus leading to false negative calls and misdiagnosis. To address these questions and evaluate the effect of the cell cycle phase on the detection of chromosomal aberration, cell lines with known imbalances were grown in log phase and flow sorted to select S- and G0/G1-phase cells and the copy number profiles of individual cells were analyzed by aCGH.

## Results

In order to study the effect of the cell cycle phase on the specificity and sensitivity of copy number aberration detection we used cell lines with known chromosomal imbalances. Two EBV cell lines with a 25.16 Mb interstitial duplication of 7p and an unbalanced translocation, with a 24 Mb duplication of 9p and an 8 Mb deletion of 18p, respectively, as well as one fibroblast cell line with a 9.3 Mb duplication of 18p and a 1.7 Mb deletion of 20p, due to an unbalanced translocation were selected to cover a range of different aberrations of various sizes. After sorting of the different cell cycle phases by flow cytometry, the genomic content of populations of cells, as an internal control for the sorting procedure, as well as individual S- and G0/G1-phase cells were analyzed by aCGH using 24Sure + BAC arrays, the most commonly used arrays for PGD purposes. An imbalance was regarded to be reliably detected in its full length when the value of log2 intensity ratio passed the threshold of +0.3 for gains or -0.3 for losses for at least half of the probes in the region of interest with a minimum of 10 consecutive probes meeting this criterion.

### Populations of S-phase cells result in more scattered aCGH profiles compared to G0/G1-phase cell populations

**Figure 1:**
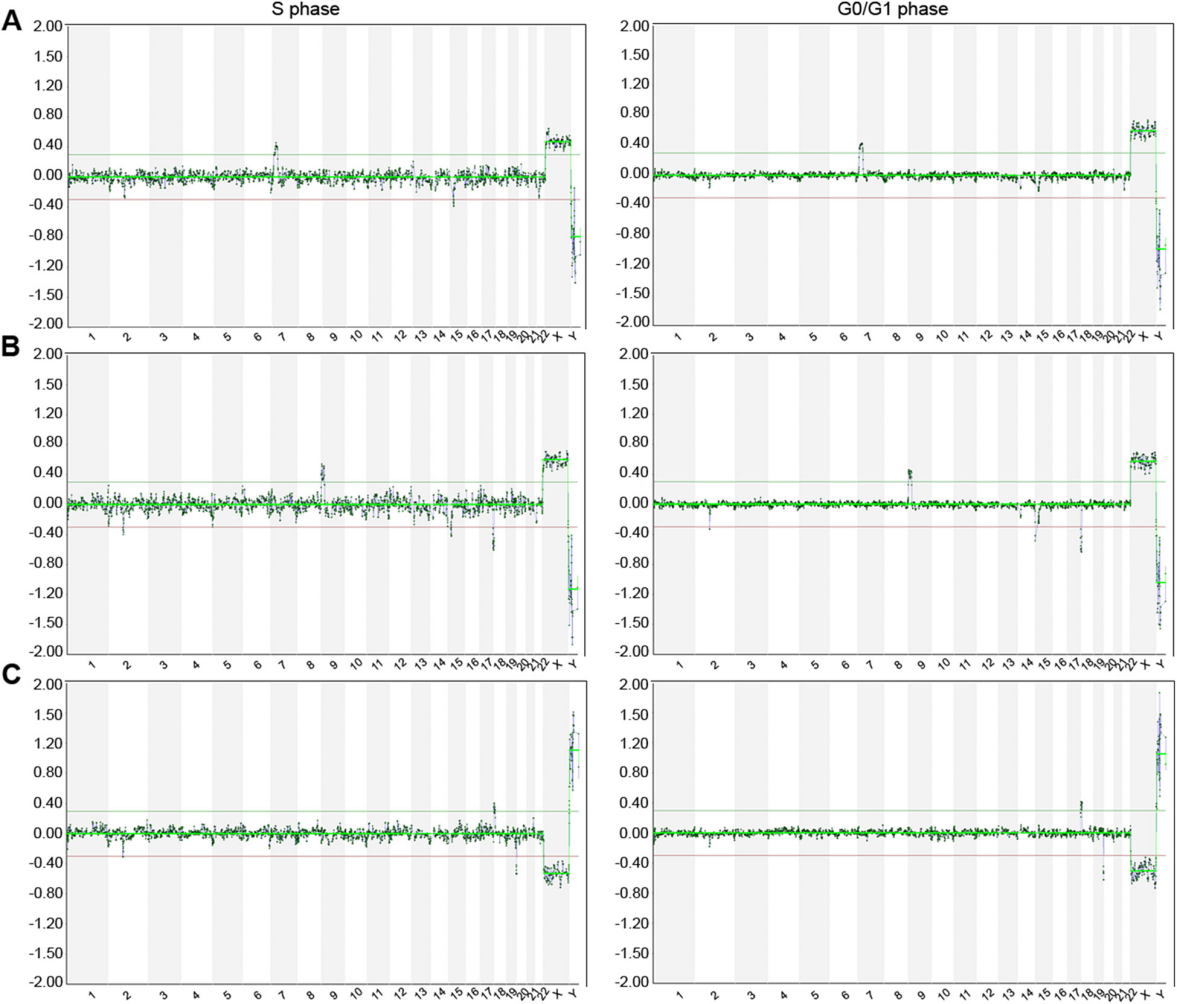
**aCGH using DNA isolated from S- and G0/G1- populations of three cell lines.** aCGH plots of genomic samples with chromosomes on the X-axis and fluorescence intensity log2 ratios on the Y-axis deriving from S- (left) and G0/G1-phase (right) sorted subpopulations of multiple cells for the three tested cell lines. **(A)** dup(7)(p14.3p21.3), **(B)** der(18)t(9;18)(p21.3;p11.3) and **(C)** der(20)t(18;20)(p11.22;p13).

To compare the aCGH profiles of S- and G0/G1-phase cells and as an internal control for the successful separation of the corresponding subpopulations by FACS, we hybridized genomic DNA extracted from many cells of S- and G0/G1-phase populations against control DNA of the opposite sex using BAC arrays. As depicted in Figure 1, the aCGH profiles produced by the two distinct subpopulations of cells differ considerably. In the plots deriving from the G0/G1 subpopulation, we can clearly see the smooth profile expected by genomic samples. On the contrary, S-phase subpopulations result in a more oscillating profile characterized by remarkably deviating log2 intensity ratios throughout the genome.

### The replication profile of the sorted S- and G0/G1-phase cells matches the expected profile according to known replication domains

To confirm the cell cycle phase identity of the isolated cells, we analyzed the log2 values of the cells in light of the known replication domains for the cell type, as described in our previous study [25]. In short, we detected an oscillation in the log2 ratios across the genome of the S-phase cells, in line with the known replication timing. In S-phase cells, regions replicating early in S-phase demonstrate high log2 ratios and late replicating regions demonstrate low log2 ratios, while in G0/G1-phase cells this wave is absent (average G0/G1 = 0,13; average S = 0,27; Mann Whitney: p < 0,0001) (*See* Additional file 1). The log2 intensity ratios of the S-phase cells also showed a significantly higher correlation to the cell type- and locus- matching replication factor values obtained from multi-cell DNA studies [28,29] when compared to G0/G1-phase cells.

For 33 out of the 35 single EBV cells and for 11 out of the 13 fibroblasts, the cell cycle phase attributed by the presence or absence of oscillation in the log2 ratios across the genome according to the replication timing confirmed the cell cycle phase of the flow sorted fraction (*See* Additional file 1). The cells not matching these criteria for S- or G0/G1-phase cells could be attributed to either an inaccuracy in the FACS experiment, natural progression of the cell in between the FACS and the cell lysis, or substandard amplification or hybridization leading to the underlying patterns.

### S phase interferes with the detection of chromosomal aberrations

**Figure 2:**
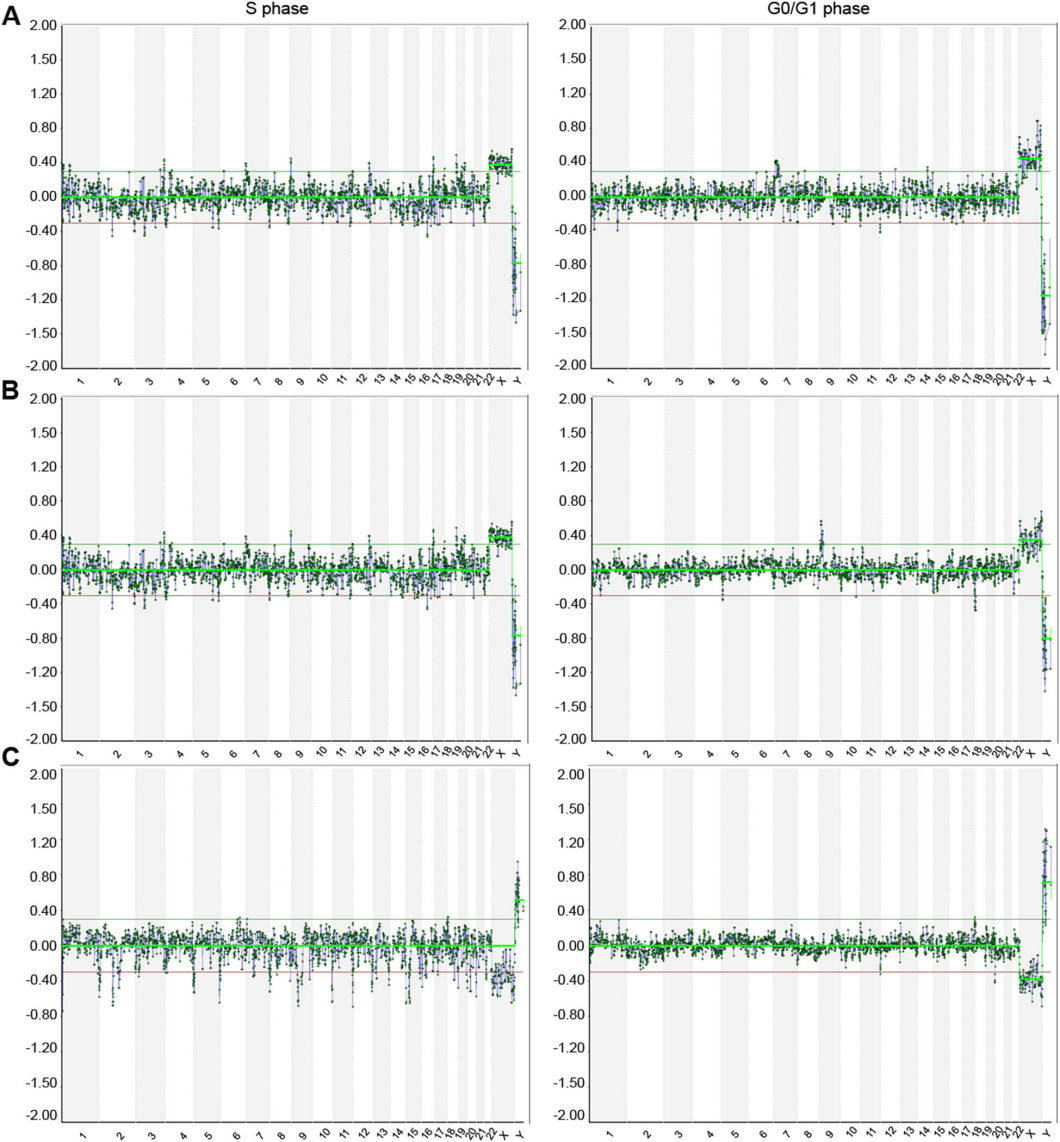
**aCGH using whole genome amplified DNA from single S- and G0/G1-phase cells of three cell lines.** aCGH plots of single cell samples with chromosomes on the X-axis and fluorescence intensity log2 ratios on the Y-axis. Representative S- (left) and G0/G1-phase (right) sorted cells for the three tested cell lines, **(A)** dup(7)(p14.3p21.3), **(B)** der(18)t(9;18)(p21.3;p11.3) and **(C)** der(20)t(18;20)(p11.22;p13), are shown.

To assess the sensitivity of the detection of copy number alterations in S- and G0/G1-phase cells we evaluated the frequency of calling of the known aberrations. We hybridized WG amplified single-cell S- and G0/G1-phase samples against control WG amplified DNA of the opposite sex, using BAC arrays. Representative examples of the analysis of the aCGH data are shown on Figure 2, while all the results for the chromosomes of interest are shown on Additional files 2, 3, 4, 5, 6 and 7. To more specifically estimate the impact of false positive calls in S-phase cells in the regions of interest, we counted the number of the BAC probes that detected an imbalance within these regions in the cell lines where there are no such aberrations. The results are summarized in Additional file 8. In short, the number of probes passing the threshold of 0.3 is quite low and in most cases remains beneath 50% of the probes in the region of interest, a number that would not have been efficient for the detection of an imbalance. Nevertheless, there are a few cells in which the number of probes passing the threshold are within the same range as the S phase cells of the cell line carrying the aberration of interest. More specifically, in 4/16 S-phase cells from the cell lines used as controls, 8 to10 probes (out of the 40 probes in the region of interest) are passing the threshold, indicating the presence of a duplication on 7p.

#### Cell line 46,XX,dup(7)(p14.3p21.3)

There are 40 BAC clones covering the 25.16 M duplicated region on 24Sure + arrays (RP11-172O13 – RP5-1143H19). Seven G0/G1- and 10 S-phase cells were selected for analysis based on the WGA and aCGH quality. In 1 G-phase sample, cell G0/G1_1_3, less than half of the probes indicated a log2 intensity ratio of a duplication, where the distal end of the duplication was weakly indicated by the elevated log2 intensity ratio of only two probes. However, in contrast to G-phase cells, there were several detection inaccuracies in the 10 S-phase cells that were analyzed (Table 1). Only in 2 of them (S_1_1 and S_1_10) the duplication was accurately called due to the elevated log2 intensity ratios of more than half of the BAC clones. In the remaining 8 samples, the calling threshold was passed by less than half of the probes in the region of interest, and notably in 6 (S_1_2, S_1_3, S_1_5, S_1_6, S_1_8 and S_1_9) more than 2/3 of the probes failed to do so, leading to underestimation or even failed detection (S_1_9) of the duplication. Additionally, in 5 of these samples (S_1_2, S_1_3, S_1_5, S_1_8 and S_1_9), the intensity log2 ratios for more than 10 consecutive probes did not pass the threshold for the detection of the duplication at one side of the duplication.

**Table 1:** Detection of the derivative chromosomes in single S- and G0/G1 cells of three cell lines

**A**							
	**dup(7p) 25.16 Mb (40 probes)**		**dup(9p) 24 Mb (42 probes)**	**del(18p) 8 Mb (28 probes)**		**dup(18p) 9.3 Mb (30 probes)**	**del(20p)**^**a **^**1.7 Mb (8 probes)**
**S_1_1**	25	**S_2_1**	26	3^a^	**S_3_1**	7	7
**S_1_2**	11	**S_2_2**	21	13	**S_3_2**	14	7
**S_1_3**	13	**S_2_3**	19	21	**S_3_3**	4^a^	6
**S_1_4**	18	**S_2_4**	29	20	**S_3_4**	16	7
**S_1_5**	8^a^	**S_2_5**	22	25	**S_3_5**	11	6
**S_1_6**	9^a^	**S_2_6**	25	18	**S_3_6**	13	8
**S_1_7**	18	**S_2_7**	28	21			
**S_1_8**	13	**S_2_8**	27	22			
**S_1_9**	10^a^	**S_2_9**	18	24			
**S_1_10**	27	**S_2_10**	31	22			
**G0/G1_1_1**	23	**G0/G1_2_1**	16	13	**G0/G1_3_1**	2^a^	7
**G0/G1_1_2**	20	**G0/G1_2_2**	19	17	**G0/G1_3_2**	17	6
**G0/G1_1_3**	18	**G0/G1_2_3**	25	19	**G0/G1_3_3**	15	7
**G0/G1_1_4**	28	**G0/G1_2_4**	36	23	**G0/G1_3_4**	19	5
**G0/G1_1_5**	24	**G0/G1_2_5**	31	17	**G0/G1_3_5**	17	6
**G0/G1_1_6**	20	**G0/G1_2_6**	23	20	**G0/G1_3_6**	17	7
**G0/G1_1_7**	25	**G0/G1_2_7**	22	20	**G0/G1_3_7**	23	4
		**G0/G1_2_8**	31	19			
**B**							
	**Underestimation**^**b**^	**No call**^**a**^	**Total number of false negative**		**Total number of false positive**	**Total number of cells**	
**S**	14	3	17		5^c^	26	
**G0/G1**	3	1	4		0	22	

**(A)** Number of probes indicating the presence of an imbalance in the regions of interest according to the BAC array log2 intensity ratios of single cells per cell phase and cell line. In brackets the number of probes in the regions of interest. The number of probes indicating the presence of the imbalance of interest was significantly higher in G0/G1- compared to S-phase cells (Mann Whitney p = 0.029). **(B)** Total number of miss-calls per cell cycle phase. The accurate imbalance detection rate is significantly higher in G0/G1- compared to S-phase cells (Chi-square test p = 0.001).

^a^The imbalance was not automatically indicated by the software.

^b^The proximal or distal end of the imbalance was very weakly detected or not detected at all.

^c^The log2 intensity ratios for 1 cell were indicative of a deletion in the control sample, while there is a duplication in the same region in the cell line of interest.

#### Cell line 46,XX,der(18)t(9;18)(p21.3;p11.3)

There are 42 and 28 BAC clones covering the 24 Mb duplication on 9p (RP11-143M1 – RP11-149I2) and 8 Mb deletion on 18p (RP11-683L23 – RP11-162D20), respectively. From the 8 G0/G1-phase cells that were analyzed, the imbalances were indicated by the altered log2 intensity ratios of more than half of the probes in 6. For cell G0/G1_2_2, the 9p duplication was marginally detected by 19/42 probes, while in the case of cell G0/G1_2_1, which was also highlighted by the replication domain analysis as deviating from the normal profile of G-phase cells, both imbalances were poorly called. On the contrary, there were 4 out of the 10 S-phase cells of this cell line, where the presence of one of the imbalances was indicated by altered log2 intensity for less than half of the probes (Table 1). More specifically, the 9p duplication was underestimated in cells S_2_9 and S_2_3, where the proximal end of the duplication was very weakly detected or not detected at all, respectively. The deletion of 18p was underestimated in S_2_2 and not detected at all in S_2_1, where the diminished log2 intensity ratios for only 3 probes in the region of interest were indicative of the deletion.

#### Cell line 46,XY,der(20)t(18;20)(p11.22;p13)

This cell line carries smaller imbalances. There are 30 BAC probes covering the 9.3 Mb 18p duplication (RP11-683L23 – RP11-36J15) and only 8 cover the 1.7 Mb 20p deletion (RP11-530N10 – RP11-77C3). Despite the size of this deletion, it was possible to discern the presence of the imbalance in both S- and G-phase cells. The log2 intensity ratio threshold for the detection of the 18p duplication was passed for more than half of the probes in 6 out of 7 G-phase cells that were analyzed, with the exception of cell G0/G1_3_1 which was also previously detected as deviating from typical G-phase cell profiles through the replication domain analysis. In contrast to that, the log2 intensity ratios of less than half of the probes covering the duplicated region passed the threshold for the detection of the duplication in 5 (S_3_1, S_3_2, S_3_3, S_3_5 and S_3_6) of the 6 S-phase cells that were analyzed (Table 1). The effect was even more profound for 2 of those cells (S_3_1 and S_3_3), where less than 1/3 of the intensity ratios of less than 1/3 of the probes were above the detection threshold, fact that could even lead to no detection of the imbalance in the case of cell S_3_3.

The analysis of all S- and G0/G1-phase cells showed no statistically significant difference in the number of probes passing the log2 ratio threshold indicating the presence of the imbalance of interest between the two groups (Mann Whitney p = 0.371). The analysis of the larger imbalances though (>9 Mb), that can also be more accurately detected by the 24Sure + array, showed a statistically significant correlation of the number of the probes indicative of the presence of the imbalance of interest and the cell cycle phase of the cell, with less probes passing the log2 ratio threshold of 0.30 in S- compared to G0/G1-phase cells (See Table 1A, Mann Whitney p = 0.029). Moreover the accurate imbalance detection rate is significantly higher in G0/G1- compared to S-phase cells (See Table 1B, Chi-square test p = 0.001), leading in higher false negative rates in S phase.

## Discussion

PGD is offered to couples that carry balanced translocations and wish to have healthy children. aCGH is becoming the standard approach for the detection of structural DNA imbalances has turned aCGH into the gold standard in the field. Current methodology does not take into account the cell cycle phase of the analyzed cell. Here we demonstrate that the results acquired after the analysis of S-phase cells differ from those of G-phase cells and we show that the accuracy of single cell copy number profiling is cell cycle dependent. In conformity with our previous work, we report increased false positive CNV detection genome-wide in S-phase cells (Figure 2). The variability in DNA-replication status for consecutive loci across the genome of S-phase cells can not only lead to false positive, but more importantly also to false negatives for structural DNA imbalances. In contrast to G-phase cells, where the imbalances were efficiently detected, we revealed that 17 out of the 26 S-phase cells where less than half of the probes in the regions of interest indicated the presence of the aberration, leading to underestimation of the size of the imbalance, or even no detection of at least one of the imbalances in 3/26 S-phase cells. Here we show that in S-phase cells CNV calling by aCGH is less sensitive.

A total of 3 misdiagnoses for chromosomal rearrangements have been reported after 2731 embryo transfers, giving a low misdiagnosis rate of 0.1% [30]. High-quality array profiles are required to study the integrity of single cell genomes with maximum possible sensitivity and specificity. The concept of the cell cycle dependent accuracy has already been mentioned by Pujol *et al.*[27]. They reported that the use of cells in S phase for PGD by FISH could lead to misdiagnosis in 7% of the cells due to presence of double dots in replicating chromatin domains and misinterpretation of the result [27]. More recently, it was also reported that the analysis of array data from fast dividing cells can be challenging due to oscillating array-CGH patterns. These patterns could be explained by the presence of a high proportion of replicating cells in S phase, especially if a specific clone prevails [31]. We previously demonstrated that the analysis of single cell genomes by array CGH is indeed challenging and can be affected by the cycle stage of the cell [25].

Even when performing stringent quality controls at each step of the aCGH-based PGD procedure, from biopsy, DNA amplification and quality, labeling efficiency, hybridization performance, image scanning, and data extraction and analysis, the analysis of S-phase cells could lead to unsatisfactory and low quality data. The analysis of such data can result in false-positive detection of CN alterations or masking the identification of existent CN imbalances. False positive CN calls could easier occur leading to the rejection of a normal embryo for transfer. False negative CN calls, on the other hand, can be even more dangerous, as they could lead to misdiagnosis in case an abnormal embryo is transferred. Fortunately, the detection of an unbalanced embryo is usually based on the detection of more than one segments [12]. Nevertheless, the detection of imbalances, especially of gains and smaller losses, as single events could be more challenging. Here we show that duplications varying from 9 to 25 Mb and deletions of 8 Mb can be missed in S phase cells. Consequently, it would be recommendable to use G0/G1-phase blastomeres for PGD purposes.

In search for a solution for the false negative calls we tried to bioinformatically correct for them (data not shown). The correction against the recurrent fluctuation that corresponds to specific replicating domains in S phase would hopefully lead to more clear copy number profiles and would finally unmask the aberrations of interest. Unfortunately, this was not the case. We did not manage to improve the detection sensitivity and specificity after the computational correction algorithm [32] that we applied. It is possible that new algorithms more powerful and suitable for these purposes could be developed in the future. One could argue that increasing the number of probes in the region of interest could consequently increase the specificity and sensitivity of the aneuploidy calling. Taking into account that the typical fluctuation observed in aCGH profiles of S-phase cells is not due to technical aspects, but the result of an innate characteristic of the DNA of cells in S phase, we would not expect the increase in the number of probes to overcome the problem. A possible solution could be the selection of cells which are not in S phase. One approach might be the use of a cell cycle phase-specific marker indicating the cell stage in which the nucleus analyzed is found [27]. Another approach may be the use of live imaging systems for monitoring the morphokinetics of the embryo development, which will enable us to follow the embryo development in real time [33]. Embryo biopsy could then be performed immediately after a cell division event, ensuring this way that the cell to be analyzed will be in the G phase of its cell cycle. Finally, the use of blastocyst stage biopsy could possibly lead to less S-phase-biased results. Given that the DNA of more than one cells is analyzed and the possibility that the 5–10 biopsied trophectoderm cells are in different cell cycle phases, we could expect improved, less fluctuating profiles.

Although the discussion regarding the use of SNP arrays and more recently single cell massive parallel sequencing (MPS) for PGD purposes has been going on for some time now, their application is still in primary stage and aCGH remains the gold standard. Nevertheless, for the future implementation of these techniques in the clinic, it should be kept in mind that the same problems regarding the analysis of S-phase cells will be faced.

## Conclusions

Our results demonstrate that the accuracy of segmental chromosomal imbalances detection by aCGH is cell-cycle dependent and, more specifically, reduced in S-phase single cells. In S phase, logR intensity ratios are remarkably variable and at the same time the frequency of false negative calls are increased in aCGH plots. Consequently, this quality compromise observed in aCGH data from S-phase cells could be a source of misdiagnosis in PGD. Hence, the cell cycle phase of the analyzed cell is of great importance and should be taken into account during the analysis. This knowledge may guide future technological improvements.

## Methods

### Cell lines and cell culture

Established Epstein-Barr virus (EBV) immortalized lymphoblastoid cells and immortalized fibroblasts were grown in 75-cm^2^ plastic flasks (BD Falcon, USA) under standard culture conditions in Dulbecco's Modified Eagle Medium/Nutrient Mixture F-12 (DMEM/F12) (Gibco, USA) medium complemented with 10% Fetal Bovine Serum (FBS) (Thermo Scientific, USA). All cell lines carried known structural chromosomal aberrations. More specifically, two EBV cell lines with a duplication and an unbalanced translocation, respectively, and one fibroblast cell line with an unbalanced translocation were used. The first cell line was derived from a female 46,XX,dup(7)(p14.3p21.3) carrier of a 25.16 Mb duplication, the second had a karyotype 46,XX,der(18)t(9;18)(p21.3;p11.3) leading to a 24 Mb duplication on the short arm of chromosome 9 and an 8 Mb deletion on the short arm of chromosome 18 and the third one was a 46,XY,der(20)t(18;20)(p11.22;p13) cell line with a 9.3 Mb duplication on the short arm of chromosome 18 and a smaller deletion of 1.7 Mb on the short arm of chromosome 20.

### Cell staining and Fluorescent activated flow cytometry

Live cells were stained with Vybrant DyeCycle Orange stain (Invitrogen, USA) according to the manufacturer’s protocol. In short, cells were washed and diluted to a final concentration of 10^6^ cells/ml in 1xPBS (Gibco/Invitrogen, USA) and consequently stained with 2 μl/10^6^ cells Vybrant DyeCycle Orange at 37°C for 30 min. Fluorescence-activated cell sorting (FACS) of all 3 cell lines for the distinction and collection of the three different cell cycle phases followed, using FACSAria III with FACSDiva software (BD Biosciences, CA). The sorting parameters were strict (single cell selection only, low collection rate) and the window of selection as narrow as possible, to ensure the highest possible purity of the isolated subpopulation (*See* Additional file 9).

### Isolation of cell subpopulations and individual cells and whole genome amplification

Cell subpopulations in S- and G0/G1-phase were collected from cultured cell populations after FACS. Sorted cells were individually picked using a mouth pipetting system as described [34]. Single cells were separately collected per cell phase per cell line and single cell whole genome amplification (WGA) was performed using the Sureplex amplification system (BlueGnome, UK) according to manufacturer’s protocol. WGA products were purified using the High Pure PCR Product Purification Kit (Roche, DE).

Fractions of 1-2×10^6^ of S- and G0/G1-phase cells were also collected, lyzed by overnight incubation at 50°C in lysis buffer (0.25 mM EDTA, 0.5% SDS and 0.1 mg/ml proteinase K in 1×PBS), and DNA was extracted using standard phenol/chloroform extraction and ethanol precipitation.

Several steps of quality assessment of the samples were applied. First the quality and quantity of the WG amplified DNA was assessed. The concentration as well as the quality of amplified and genomic DNA was evaluated by nanodrop spectophotometer (ND-1000, Nanodrop Technologies, USA). Only the single cells that gave an amplification product of 2 μg or more were further used for hybridization on an array. No amplification (n = 1) or low yield (n = 5) of WG amplified DNA could be attributed to the non-transfer of a cell in the tube or to transfer of cellular fragments instead of good quality intact cells. Samples with bad 260/280 and 260/230 ratios (n = 2) were also not further used.

### BAC array hybridization and analysis

Array CGH for both genomic and single cell whole genome amplified samples was carried out using 24sure + V1.0 BAC Cytochip microarrays, the most commonly used arrays in PGD for the detection of unbalanced translocations and subchromosome imbalances, as they are enriched with more probes covering the pericentromeric and subtelomeric regions. Four different batches of 24sure + V1.0 BAC arrays (BlueGnome, UK) were used following the standard protocol (BlueGnome 24sure protocol, http://www.cytochip.com). For the hybridizations, 200 ng of sample DNA and equal amount of commercially available reference DNA of the opposite sex (sex mismatch experiments) were labeled for 2 h by random primer labeling (BlueGnome, UK) using Cy5- and Cy3-dCTPs, respectively. For multi-cell samples, commercially available reference male or female DNA (Kreatech, NL) was used, while for the hybridizations with single-cell amplified material, SureRef male or female reference DNA (BlueGnome, UK). Hybridization and washing was performed according to the manufacturer’s protocol.

Scanning was performed using a DNA-microarray scanner (Agilent Technologies, UK), the separation of the duplex raw images by ImageViewer and the analysis of the data by BlueFuse v3.0 software (BlueGnome, UK). The evaluation of the array data and threshold settings to call an abnormality were performed according to the guidelines of the manufacturer and samples that failed to pass these criteria (n = 4) were not used for further analysis. Gains and losses were called when the log2 ratio of BAC clones of interest were above or below the threshold of 0.3, respectively.

### Data normalization and copy number calling

For further analysis, the raw data were feature extracted using GenePix Pro 6.0 software (Axon, Molecular Devices). The data were normalized such that the ratio of the means for all features would equal to 1. The raw grp files produced after normalization and feature extraction were used as input for R scripts (R version 2.13.2 – http://www.r-project.org) using different packages [35-37]. Log2 intensity ratios with a signal to noise less than 2 were excluded from the array. Subsequently, the data was preprocessed by the ‘Channel clone’ method described in Cheng *et al.*[32], segmented by GLAD algorithm and called by the CGHcall package in R [38,39].

### Comparison of log2 intensity ratios to predicted replication domains

For the analysis of cell cycle phase identity, the methodology as described in Van der Aa *et al.*[25] was used. Log2 values of fibroblast cells were compared to replication ratio data of IMR90 fetal lung fibroblasts (369858–3) [28] and log2 ratios of EBV cells were compared to replication ratios of C0202 lymphoblastoid cells (replicate 1, GEO accession number GSE20027) [29].

## Abbreviations

AMA: Advanced maternal age; aCGH: Array based comparative genomic hybridization; ART: Assisted reproduction technology; BAC: Bacterial artificial chromosome; CN: Copy number; ESHRE: European society of human reproduction and embryology; FACS: Fluorescence-activated cell sorting; FISH: Fluorescence *in situ* by hybridization; MPS: Massive parallel sequencing; PGD: Preimplantation genetic diagnosis; PGS: Preimplantation genetic screening; RIF: Repeated implantation failure; RM: Recurrent miscarriage; OR: Oocyte retrieval; ORI: Origin of replication; WGA: Whole genome amplification.

## Competing interests

JRV has contract research with and is advisor of Oxford Gene Technology.

## Authors’ contributions

ED performed the experiments and the data analysis and wrote the manuscript. NVDA performed the cell cycle phase determination analysis based on predicted replication domains. JC performed the copy number calling after data normalization. TV co-supervised the project. JRV supervised the experiments and drafted the manuscript. All authors read and approved the final manuscript.

## Supplementary Material

Additional file 1 **Replication profiles of the single S- and G0/G1-phase cells according to known replication domains.** (A) Plots for EBV-transformed lymphocytes and (B) corresponding plots for fibroblasts. On the left: The X-axis depicts the % GC content per probe, the Y-axis the log2 intensity ratios per sample. Each line is a Loess fit using the data of a single-cell sample, with S-phase cells shown in blue (mean in red) and G0/G1-phase cells in black (mean in green). On the right: Boxplots for single cells depicting autosomal log2 intensity ratios that were pooled per cell cycle phase and per early or late DNA-replication domain. Boxplots show the median of the log2 intensity ratios (central line) and the quartiles (box and whiskers). Click here for file

Additional file 2 **aCGH profiles of the derivative chromosomes for all the single cells analyzed.** aCGH plots of the chromosomes of interest with fluorescence intensity log2 ratios on the X-axis and chromosomal position on the Y-axis. Plots for all single-cell samples are depicted: (A) dup(7)(p14.3p21.3) G0/G1-phase cells. Click here for file

Additional file 3 **aCGH profiles of the derivative chromosomes for all the single cells analyzed.** aCGH plots of the chromosomes of interest with fluorescence intensity log2 ratios on the X-axis and chromosomal position on the Y-axis. Plots for all single-cell samples are depicted: (B) dup(7)(p14.3p21.3) S-phase cells. Click here for file

Additional file 4 **aCGH profiles of the derivative chromosomes for all the single cells analyzed.** aCGH plots of the chromosomes of interest with fluorescence intensity log2 ratios on the X-axis and chromosomal position on the Y-axis. Plots for all single-cell samples are depicted: (C) der(18)t(9;18)(p21.3;p11.3) G0/G1-phase cells. Click here for file

Additional file 5 **aCGH profiles of the derivative chromosomes for all the single cells analyzed.** aCGH plots of the chromosomes of interest with fluorescence intensity log2 ratios on the X-axis and chromosomal position on the Y-axis. Plots for all single-cell samples are depicted: (D) der(18)t(9;18)(p21.3;p11.3) S-phase cells. Click here for file

Additional file 6 **aCGH profiles of the derivative chromosomes for all the single cells analyzed.** aCGH plots of the chromosomes of interest with fluorescence intensity log2 ratios on the X-axis and chromosomal position on the Y-axis. Plots for all single-cell samples are depicted: (E) der(20)t(18;20)(p11.22;p13) G0/G1-phase cells. Click here for file

Additional file 7 **aCGH profiles of the derivative chromosomes for all the single cells analyzed.** aCGH plots of the chromosomes of interest with fluorescence intensity log2 ratios on the X-axis and chromosomal position on the Y-axis. Plots for all single-cell samples are depicted: (F) der(20)t(18;20)(p11.22;p13) S-phase cells. Click here for file

Additional file 8 **Estimation of false positive detection rate of the derivative chromosomes in control single S- and G0/G1-phase cells of three cell lines.** Number of probes falsely indicating the presence of an imbalance in the regions of interest according to the BAC array log2 intensity ratios in S-phase single cells of cell lines not carrying an aberration involving this region. In brackets the number of probes in the regions of interest. *The log2 intensity ratios were indicative of a deletion in the control sample, while there is a duplication in the same region in the cell line of interest. Click here for file

Additional file 9 **Cell sorting procedure.** Representative plot illustrating the cell sorting procedure by FACS with relative DNA content on the X-axis and the cell count on the Y-axis. The marked windows correspond to the fractions which were collected for each cell subpopulation (G0/G1-, S- and G2/M-phase). Click here for file
