# Supplementary figures and images for: Single cell segmental aneuploidy detection is compromised by S phase
Source: Mol Cytogenet. 2014 Jul 11;7:46. doi: 10.1186/1755-8166-7-46 (PMC4114140; doi:10.1186/1755-8166-7-46)

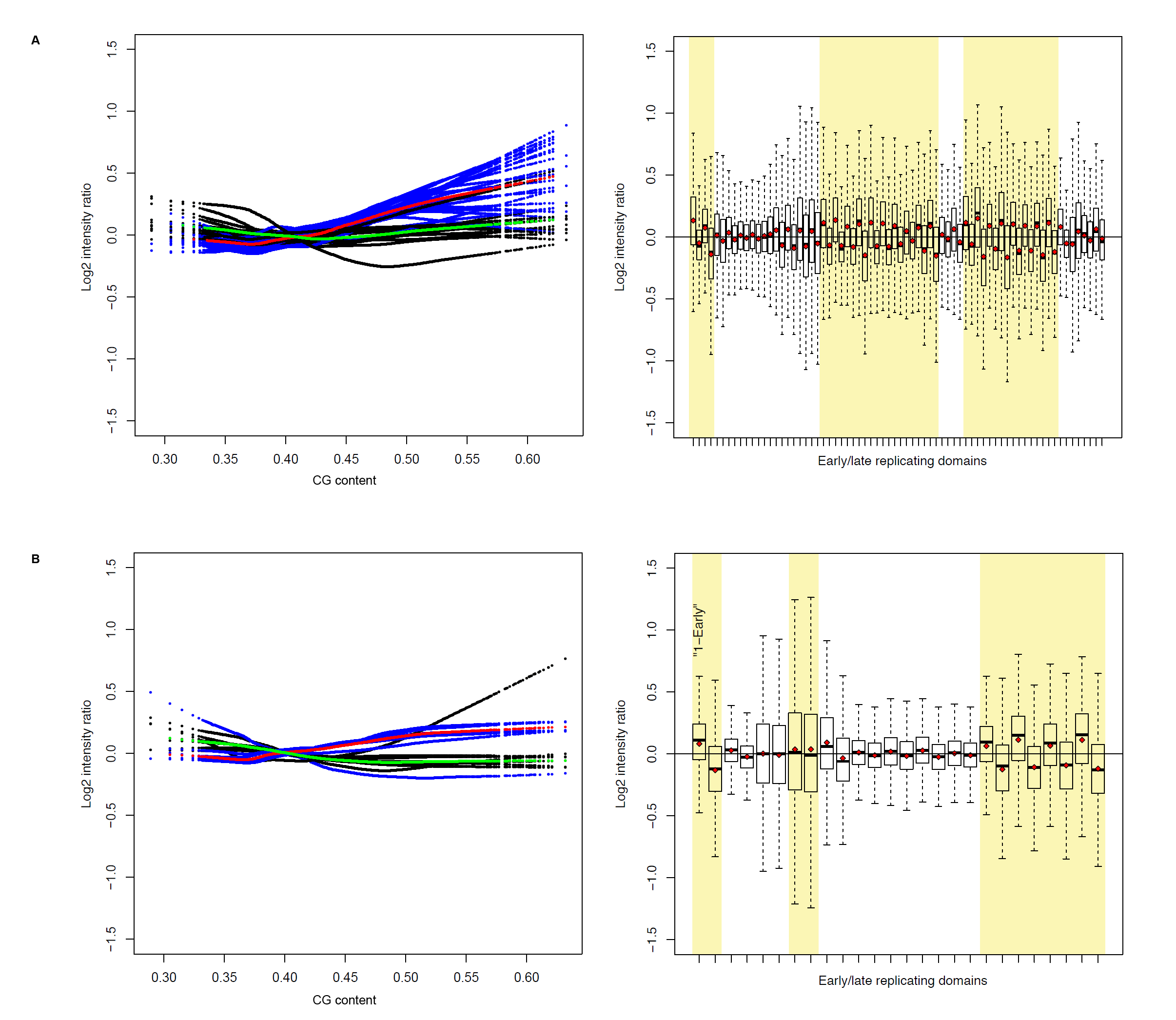

Supplement: Additional file 1 — Replication profiles of the single S- and G0/G1-phase cells according to known replication domains. (A) Plots for EBV-transformed lymphocytes and (B) corresponding plots for fibroblasts. On the left: The X-axis depicts the % GC content per probe, the Y-axis the log2 intensity ratios per sample. Each line is a Loess fit using the data of a single-cell sample, with S-phase cells shown in blue (mean in red) and G0/G1-phase cells in black (mean in green). On the right: Boxplots for single cells depicting autosomal log2 intensity ratios that were pooled per cell cycle phase and per early or late DNA-replication domain. Boxplots show the median of the log2 intensity ratios (central line) and the quartiles (box and whiskers). [file 1755-8166-7-46-S1.png]

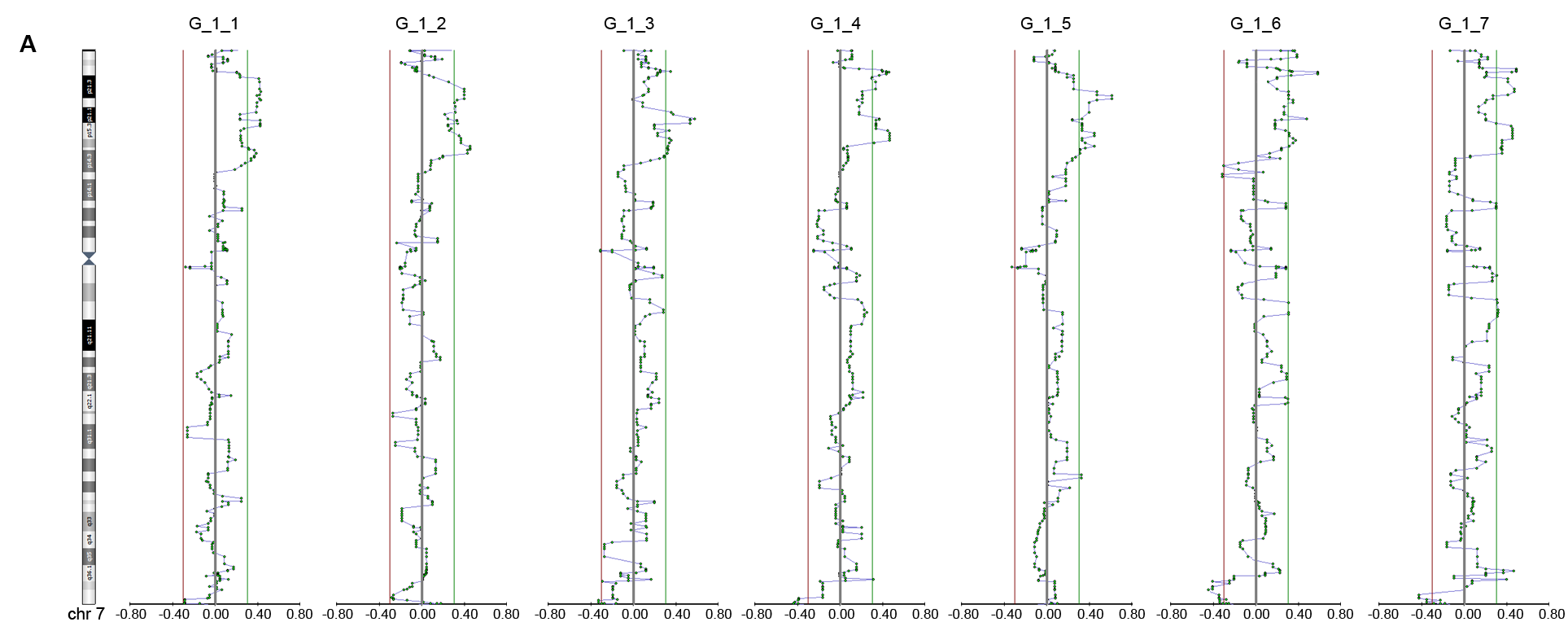

Supplement: Additional file 2 — aCGH profiles of the derivative chromosomes for all the single cells analyzed. aCGH plots of the chromosomes of interest with fluorescence intensity log2 ratios on the X-axis and chromosomal position on the Y-axis. Plots for all single-cell samples are depicted: (A) dup(7)(p14.3p21.3) G0/G1-phase cells. [file 1755-8166-7-46-S2.png]

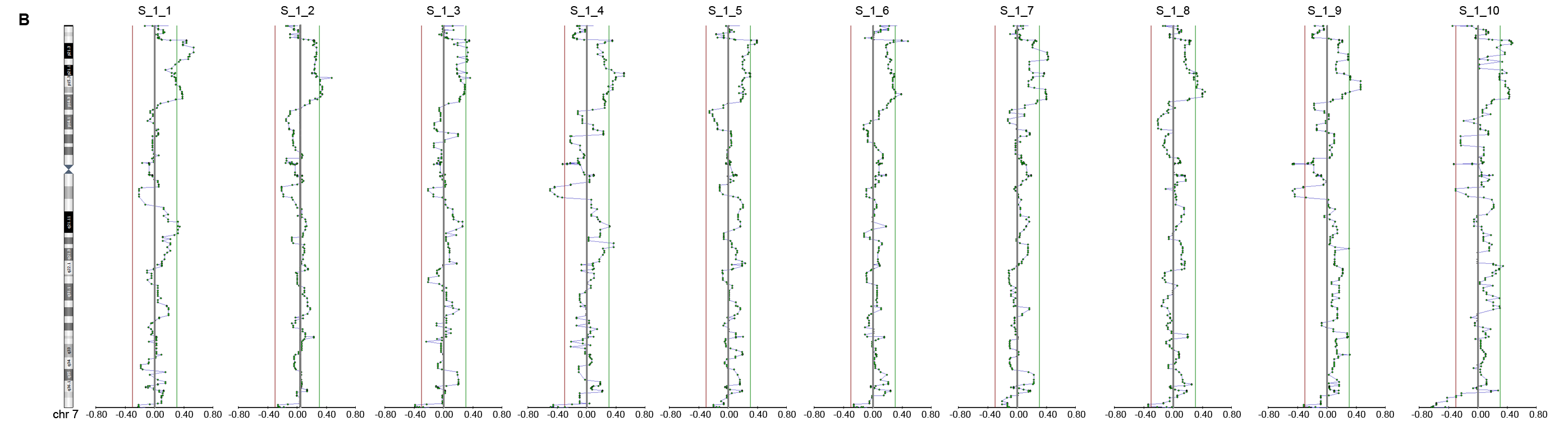

Supplement: Additional file 3 — aCGH profiles of the derivative chromosomes for all the single cells analyzed. aCGH plots of the chromosomes of interest with fluorescence intensity log2 ratios on the X-axis and chromosomal position on the Y-axis. Plots for all single-cell samples are depicted: (B) dup(7)(p14.3p21.3) S-phase cells. [file 1755-8166-7-46-S3.png]

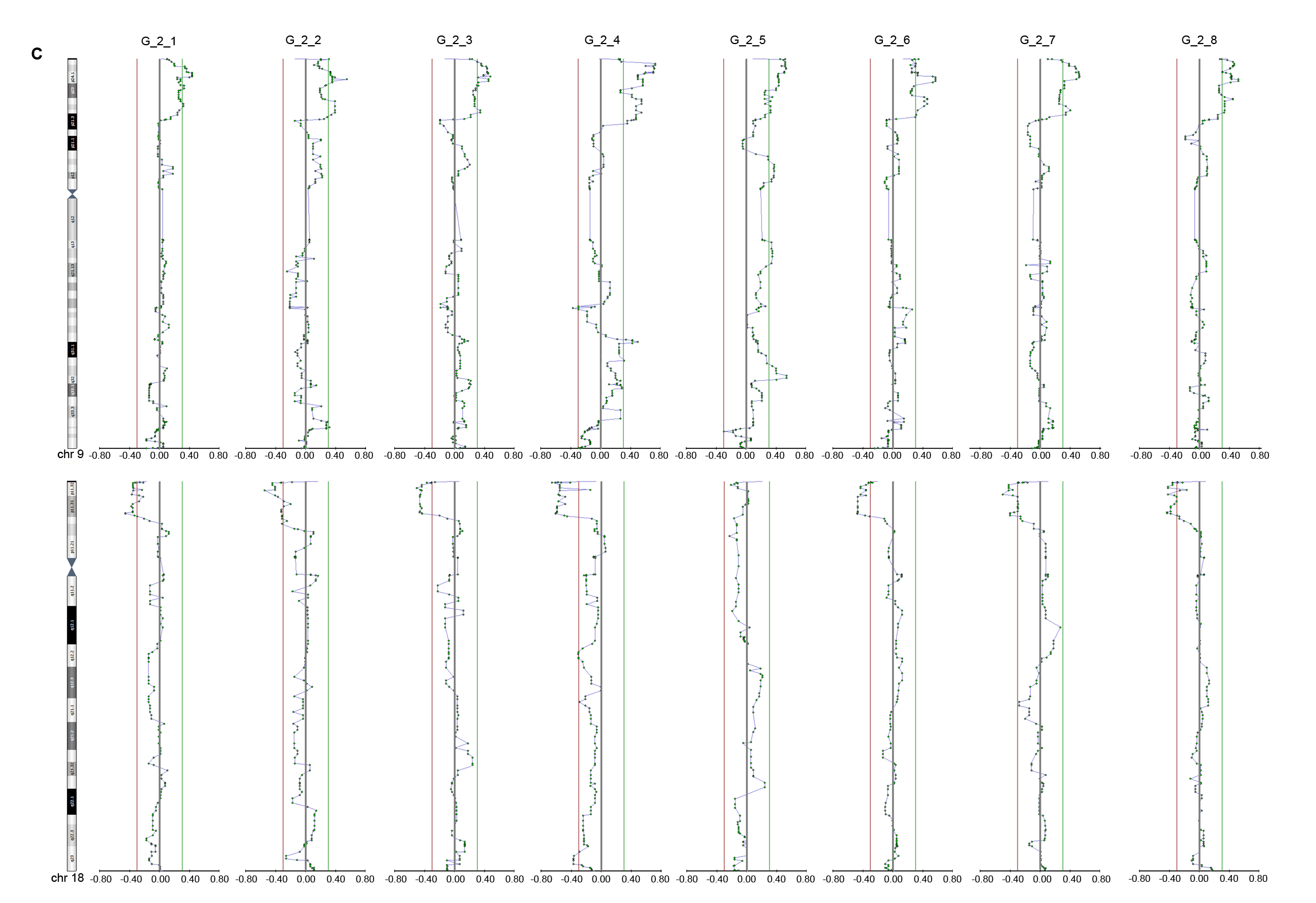

Supplement: Additional file 4 — aCGH profiles of the derivative chromosomes for all the single cells analyzed. aCGH plots of the chromosomes of interest with fluorescence intensity log2 ratios on the X-axis and chromosomal position on the Y-axis. Plots for all single-cell samples are depicted: (C) der(18)t(9;18)(p21.3;p11.3) G0/G1-phase cells. [file 1755-8166-7-46-S4.png]

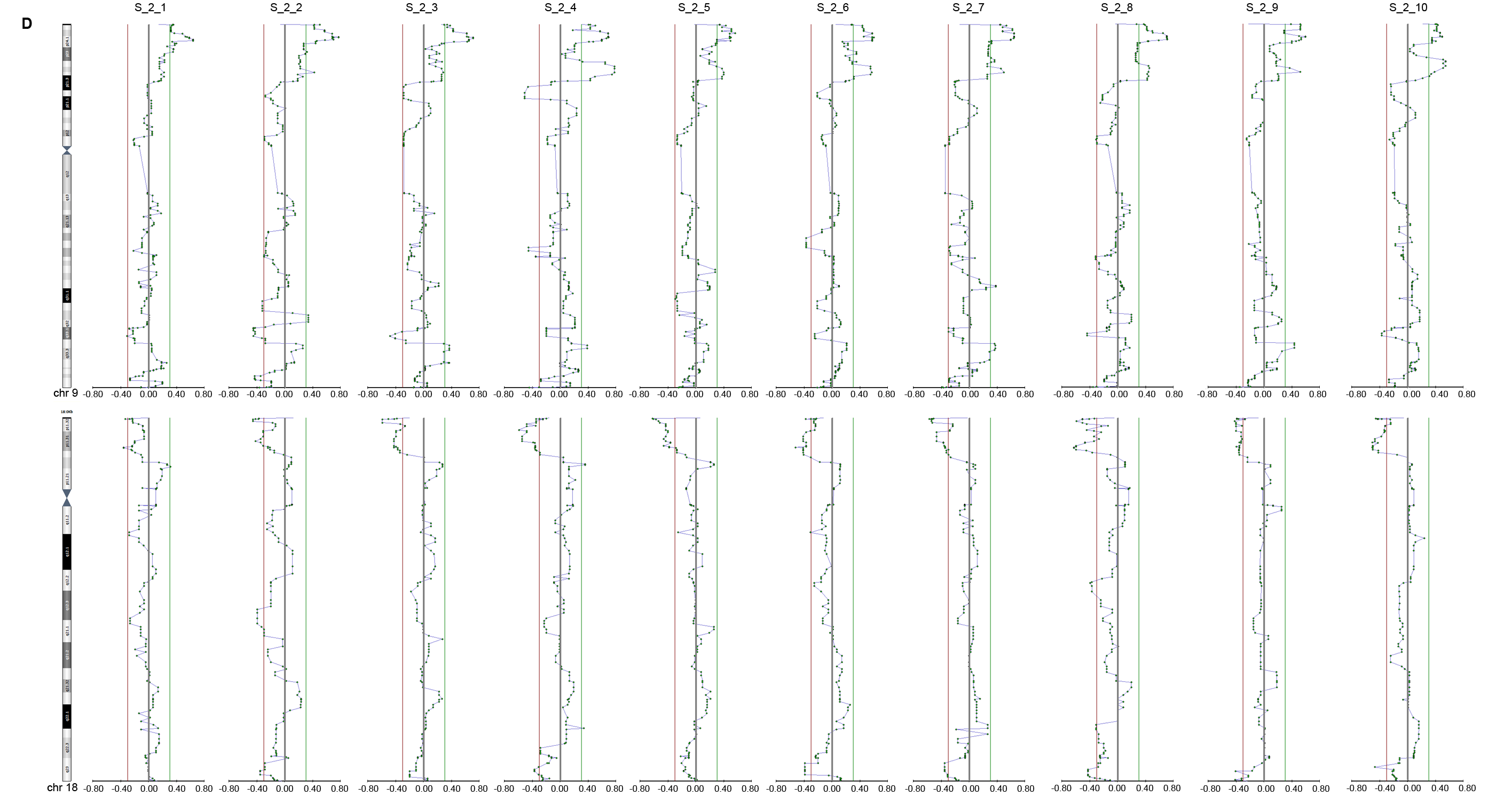

Supplement: Additional file 5 — aCGH profiles of the derivative chromosomes for all the single cells analyzed. aCGH plots of the chromosomes of interest with fluorescence intensity log2 ratios on the X-axis and chromosomal position on the Y-axis. Plots for all single-cell samples are depicted: (D) der(18)t(9;18)(p21.3;p11.3) S-phase cells. [file 1755-8166-7-46-S5.png]

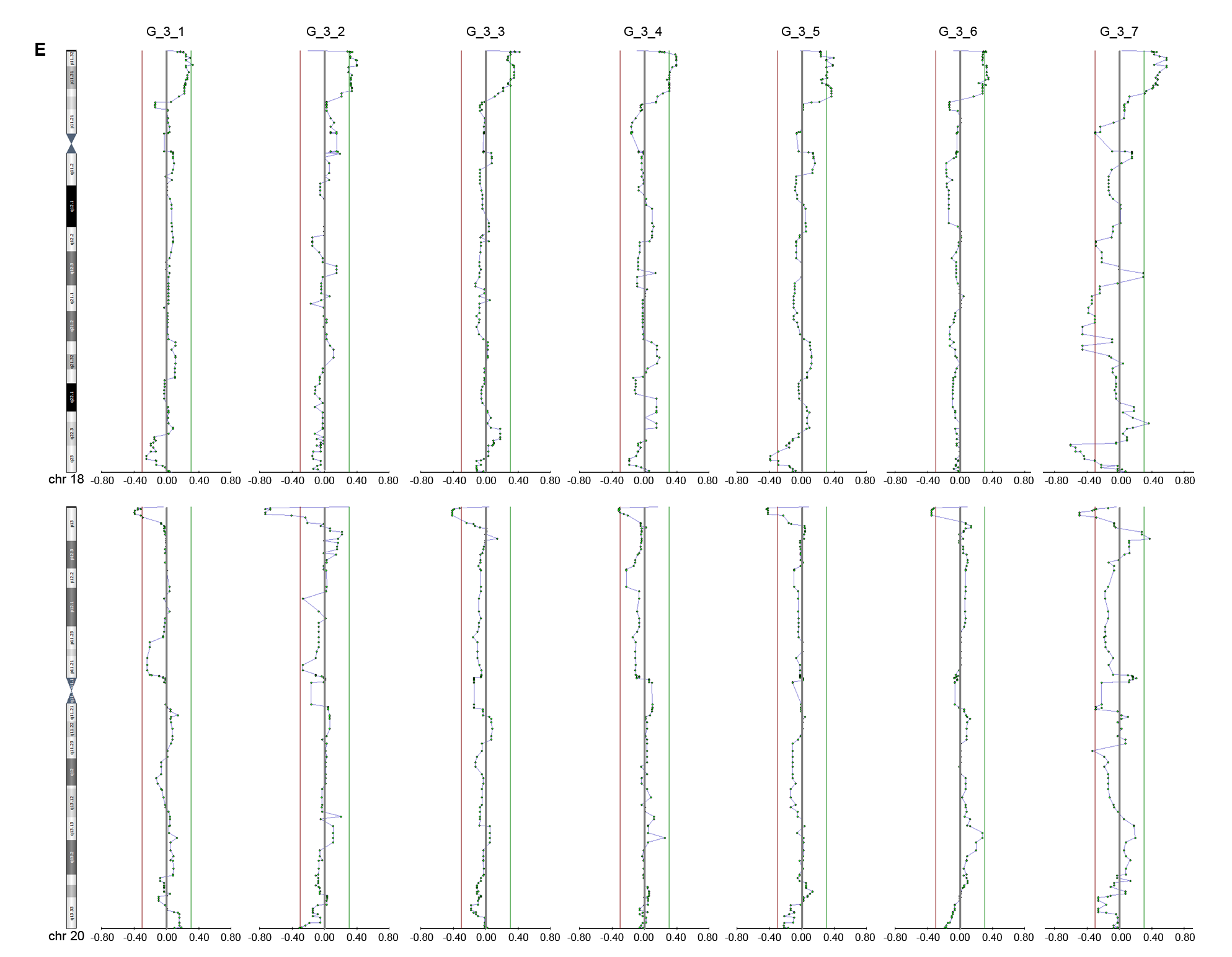

Supplement: Additional file 6 — aCGH profiles of the derivative chromosomes for all the single cells analyzed. aCGH plots of the chromosomes of interest with fluorescence intensity log2 ratios on the X-axis and chromosomal position on the Y-axis. Plots for all single-cell samples are depicted: (E) der(20)t(18;20)(p11.22;p13) G0/G1-phase cells. [file 1755-8166-7-46-S6.png]

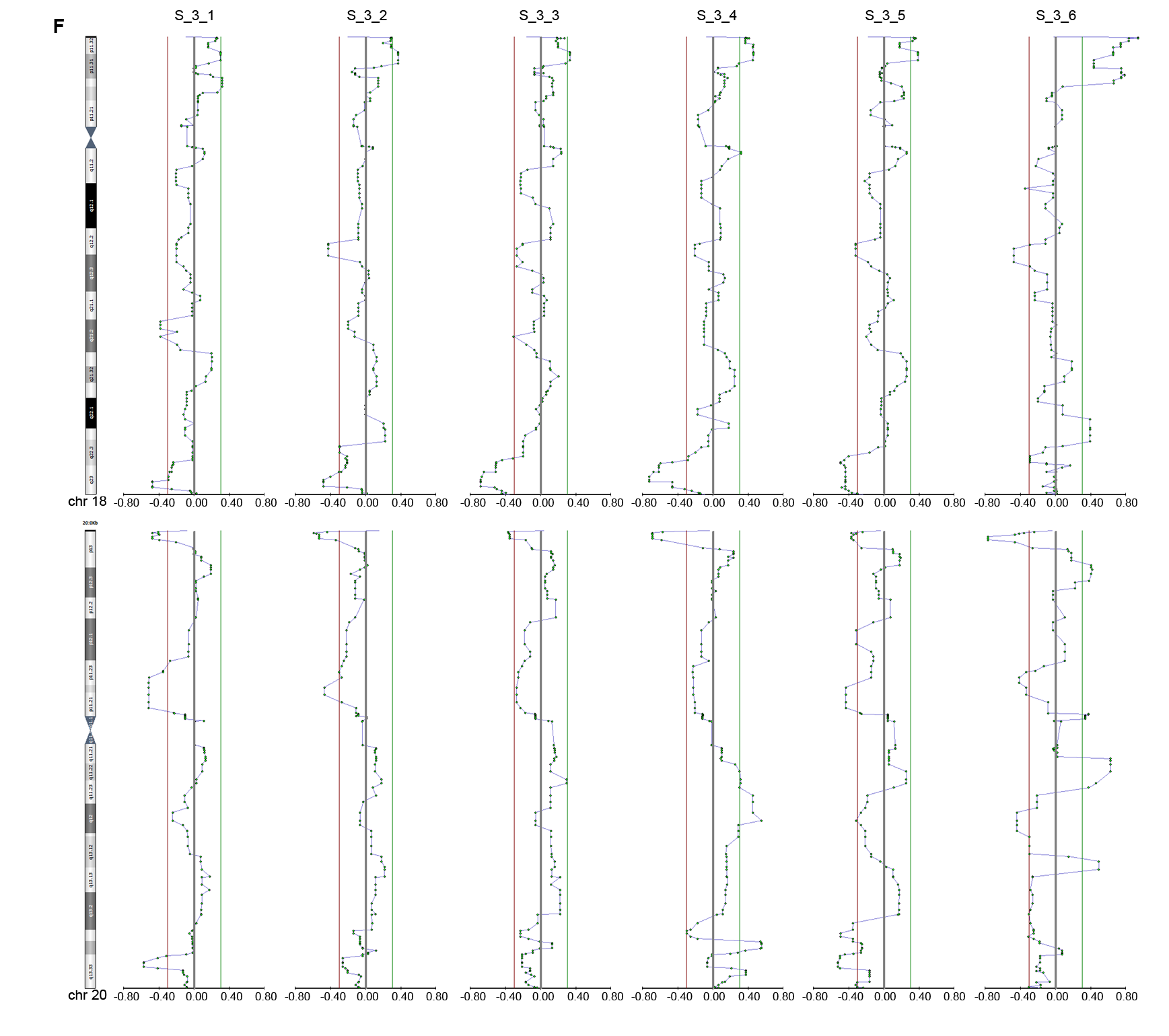

Supplement: Additional file 7 — aCGH profiles of the derivative chromosomes for all the single cells analyzed. aCGH plots of the chromosomes of interest with fluorescence intensity log2 ratios on the X-axis and chromosomal position on the Y-axis. Plots for all single-cell samples are depicted: (F) der(20)t(18;20)(p11.22;p13) S-phase cells. [file 1755-8166-7-46-S7.png]

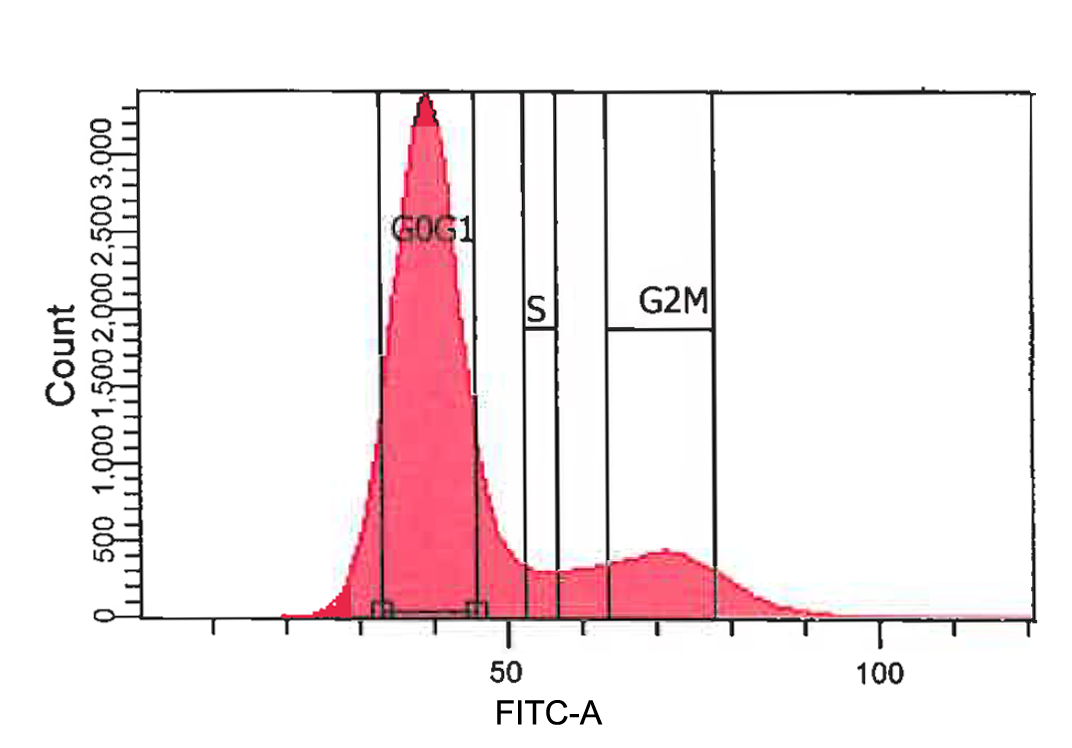

Supplement: Additional file 9 — Cell sorting procedure. Representative plot illustrating the cell sorting procedure by FACS with relative DNA content on the X-axis and the cell count on the Y-axis. The marked windows correspond to the fractions which were collected for each cell subpopulation (G0/G1-, S- and G2/M-phase). [file 1755-8166-7-46-S9.png]
